# Diet quality in pediatric migraine and the educational quality of related short-form videos: a cross-sectional study and content analysis

**DOI:** 10.3389/fnut.2026.1934926

**Published:** 2026-09-21

**Authors:** Haisu Li, Lan Liu, Jiayi Jiang, Yumei Xu, Dan Zhou, Ying Xu

**Affiliations:** 1 Department of Anesthesiology Children’s Hospital of Chongqing Medical University, National Clinical Research Center for Children and Adolescents’ Health and Diseases, Ministry of Education Key Laboratory of Child Development and Disorders, Chongqing Key Laboratory of Child Neurodevelopment and Cognitive Disorders, Chongqing, China; 2 Department of Anesthesiology, The Affiliated Dazu's Hospital of Chongqing Medical University, Chongqing, China

**Keywords:** Chinese Children Dietary Index, diet quality, dietary management, pediatric migraine, short-form videos

## Abstract

**Background:**

Diet quality has been associated with headache burden and psychosocial outcomes in children with migraine, while caregivers increasingly use short-form videos for dietary and lifestyle advice. However, evidence on overall diet quality and related video quality remains limited.

**Methods:**

This study included 180 children aged 7–18 years with migraine. Diet quality was assessed using the Chinese Children Dietary Index (CCDI). Participants were divided at the median CCDI score of 117.7 into low- and high-CCDI groups (*n* = 90 each). Between-group comparisons included Pediatric Migraine Disability Assessment (PedMIDAS) scores, headache characteristics, analgesic use, caregiver burden, and anxiety and depressive symptoms. Multivariable regression was used to examine adjusted associations between CCDI scores and clinical outcomes. Caregiver use of short-form videos was also surveyed. In addition, 150 videos on dietary and lifestyle management of pediatric migraine from Douyin, Kuaishou, and Xiaohongshu were analyzed for educational quality, reliability, and actionability.

**Results:**

The high-CCDI group had lower PedMIDAS scores than the low-CCDI group (29.54 ± 17.20 vs. 41.96 ± 14.72), fewer headache days over 28 days [6.00 (4.00, 8.00) vs. 9.00 (8.00, 11.00)], lower pain intensity, and fewer days of analgesic use (all *p* < 0.001). Caregiver burden and anxiety and depression scores were also lower (all *p* < 0.05). After adjustment, higher CCDI scores remained associated with lower PedMIDAS scores, fewer headache days, lower pain intensity, fewer days of analgesic use, and lower caregiver burden. Diet and lifestyle management was the most frequently searched topic (30.6%), yet only 34.4% of caregivers fully verified video recommendations with a physician. Although 90.0% of videos provided recommendations concordant with clinical guidelines or evidence-based recommendations, reliability, actionability, and nutrition follow-up remained limited; nutrition follow-up was addressed in only 23.33% of videos.

**Conclusion:**

Higher overall diet quality was associated with lower migraine burden and caregiver burden in children with migraine. These findings support the relevance of overall dietary assessment, while prospective studies are needed to clarify temporal and causal relationships. Short-form videos are an important source of dietary and lifestyle information for caregivers, but their reliability, actionability, and follow-up content require improvement.

## 1 Introduction

Migraine is among the most common primary headache disorders in children and adolescents, with a global age-standardized prevalence of approximately 10.3%, and is a major cause of recurrent pain, school absence, activity limitation, sleep disturbance, and impaired quality of life (1, 2). Compared with adults, children with migraine may have age-specific features, including shorter attacks, bilateral pain, abdominal symptoms, and difficulty describing headache characteristics or associated symptoms (3). These features may contribute to underrecognition and undertreatment in routine practice. Assessment of pediatric migraine should therefore consider not only headache frequency and intensity but also modifiable factors that may affect headache burden and daily functioning.

Diet and lifestyle are increasingly recognized as relevant to pediatric migraine management. Irregular meals, breakfast skipping, inadequate hydration, excessive intake of sugar-sweetened or caffeinated beverages, and certain dietary triggers may precipitate or worsen headaches in susceptible children (4, 5). However, focusing on individual dietary triggers alone may not capture broader patterns of dietary adequacy and eating behavior. Limited dietary diversity and imbalanced intakes of fruits, vegetables, dairy products, high-quality protein, and dietary fiber may reflect suboptimal dietary patterns and may influence headache burden through sleep, physical activity, body weight, and psychosocial stress (6, 7). Accordingly, assessment of overall diet quality may provide a more comprehensive perspective on nutritional and behavioral patterns in children with migraine. Diet quality indices, such as the Chinese Children Dietary Index, provide a multidimensional assessment of overall diet quality and may help identify specific nutritional and behavioral concerns in children with migraine (8). In family-based management, caregivers’ access to, understanding of, and use of dietary and lifestyle information are strongly influenced by their usual health information sources.

Short-form videos have become an increasingly important source of health information for parents and children. Their accessibility, intuitive visual format, and rapid dissemination may shape caregivers’ understanding of migraine symptoms, dietary triggers, lifestyle management, and the need for medical care (9, 10). However, the educational quality, reliability, completeness, and scientific accuracy of migraine-related videos may vary widely because they are produced by physicians, medical institutions, commercial accounts, media creators, and nonprofessional users (10, 11). Low-quality or misleading videos may overstate unproven dietary restrictions, exaggerate specific food triggers, promote inappropriate products, or provide unbalanced advice.

Evidence on dietary intake and diet quality in children with migraine in real-world clinical settings, and their associations with headache burden, remains limited. The quality of short-form video-based education on pediatric migraine and dietary management has also not been adequately evaluated. Therefore, this cross-sectional study and content analysis aimed to describe dietary intake and diet quality in children with migraine, examine the associations of overall diet quality and specific dietary components with headache burden, and evaluate the educational quality and reliability of relevant short-form videos. These findings may inform nutritional assessment, lifestyle counseling, and online health education for children with migraine.

## 2 Materials and methods

### 2.1 Study design and ethics

This single-center retrospective cross-sectional study included children diagnosed with pediatric migraine who visited the Department of Pain Medicine at the Children’s Hospital of Chongqing Medical University between January 2024 and December 2025. Clinical data and dietary intake information were retrospectively collected, and caregivers’ use of short-form video platforms was assessed through a caregiver survey. In parallel, a content analysis of short-form videos related to dietary management of pediatric migraine was conducted. Migraine burden and headache characteristics were compared between the low- and high-CCDI groups; caregivers’ short-form video use patterns were described; and the quality of relevant videos was evaluated.

The study protocol was approved by the Ethics Committee of the Children’s Hospital of Chongqing Medical University (approval number: [2026] Clinical Research Ethics Approval No. 136). In accordance with the Declaration of Helsinki, written informed consent was obtained from caregivers, and assent was obtained from participating children when appropriate.

### 2.2 Participants and short-form video selection

Children with migraine were eligible if they met the following criteria: (1) aged 7–18 years; (2) diagnosed with pediatric migraine according to the International Classification of Headache Disorders, 3rd edition (ICHD-3) (3), with the diagnosis confirmed by a pediatric pain physician experienced in headache management; and (3) had complete clinical data. The exclusion criteria were as follows: (1) secondary headache; (2) severe neurological, psychiatric, or other systemic diseases; (3) long-term use of medications that might affect headache presentation; and (4) incomplete clinical data.

For the short-form video analysis, Douyin, Kuaishou, and Xiaohongshu were selected based on the platform use reported in the caregiver survey and their widespread use as short-form video platforms in China. To minimize the influence of caregivers’ individual platform use patterns, newly registered accounts were used for systematic keyword searches during a predefined search period, following established methods for social media-based health research. The same Chinese keywords were used across all platforms: “children’s migraine diet,” “migraine nutrition management,” and “migraine dietary triggers.” Videos were screened sequentially according to each platform’s default ranking order. Ineligible videos were excluded, and screening continued until 50 eligible videos were included from each platform, yielding a total of 150 videos.

Videos were included if they met the following criteria: (1) focused on pediatric migraine, childhood headache, or diet- and lifestyle-related management of migraine; (2) provided health education, explanations, prevention strategies, dietary advice, or management recommendations; and (3) contained complete audio narration, subtitles, or sufficient textual information for quality assessment. Videos were excluded if they were: (1) duplicates; (2) purely commercial advertisements; (3) unrelated to the study topic; or (4) insufficient for quality assessment.

### 2.3 Outcome measures

Diet quality was assessed using the Chinese Children Dietary Index (CCDI) (8). Dietary intake data used to calculate the CCDI score were collected using a food frequency questionnaire. The CCDI total score ranges from 0 to 160, with higher scores indicating better overall diet quality. For clinical analysis, participants were divided into low- and high-CCDI groups according to the median CCDI total score. Children with a CCDI total score ≤117.7 were assigned to the low-CCDI group, whereas those with a score >117.7 were assigned to the high-CCDI group. Baseline characteristics, migraine burden, and headache-related clinical outcomes were then compared between groups.

The primary outcome was the Pediatric Migraine Disability Assessment (PedMIDAS) score, with higher scores indicating greater headache-related functional impairment and disease burden (12). Secondary outcomes included the number of headache days over the previous 28 days, duration of a single headache attack, days of analgesic use, and pain intensity. Pain intensity was assessed using a 10-cm visual analog scale (VAS), with higher scores indicating more severe pain (13). Caregiver burden was assessed using the 22-item Zarit Burden Interview (ZBI), with higher total scores indicating greater caregiver burden (14). Anxiety and depressive symptoms in children were assessed using the Revised Child Anxiety and Depression Scale-short version (RCADS-25), including anxiety, depression, and total scores; higher scores indicate more severe emotional symptoms (15).

Caregivers’ eHealth literacy was assessed using the eHealth Literacy Scale (eHEALS), with higher scores indicating greater ability to access, evaluate, and apply electronic health information (16). Previously validated Chinese-language versions of the Zarit Burden Interview, RCADS-25, and eHEALS were used in this study. The PedMIDAS was translated into Chinese by the study team for use in the present study. Caregiver-reported short-form video use was used to describe common patterns of obtaining pediatric migraine- and diet-related health information from short-form video platforms, including the most frequently used platform, frequency of use, average viewing duration, commonly searched topics, subjective assessment of video reliability, whether video recommendations were verified with physicians, whether video recommendations were adopted, and satisfaction with video use.

For each included video, the following characteristics and content domains were recorded: source platform, uploader type, video duration, number of likes, saves, comments, and shares, time since posting, number of uploader followers, and video content topics.

### 2.4 Short-form video quality assessment

Short-form video quality was evaluated using a multidimensional assessment framework, including the modified DISCERN tool (mDISCERN), the Global Quality Score (GQS), the Video Information and Quality Index (VIQI), and the Patient Education Materials Assessment Tool (PEMAT). The mDISCERN was adapted by Singh et al. (17) from the original DISCERN tool to assess the reliability of online health information. The GQS is a 5-point overall quality score used to evaluate the overall educational quality, flow, and usefulness of content (18). The VIQI evaluates video quality across four dimensions: information flow, accuracy, visual/production quality, and title–content consistency (precision) (19). The PEMAT assesses the understandability and actionability of patient education materials (20). Detailed scoring criteria are provided in Supplementary Tables S1–S4.

All included videos were first independently scored by one pediatric pain physician and one dietitian, both with more than 10 years of professional experience. Discrepancies were resolved through discussion. If consensus could not be reached, a third researcher with equivalent qualifications made the final decision.

### 2.5 Sample size and statistical analysis

The clinical component of this study primarily compared headache burden between the low- and high-CCDI groups; therefore, the PedMIDAS score was used as the main indicator for sample size estimation. Based on preliminary data and previous studies on pediatric migraine, the expected PedMIDAS score was 39.0 ± 18.5 in the low-CCDI group and 31.0 ± 17.2 in the high-CCDI group. With a two-sided significance level of 0.05, 80% power, and a 1:1 group allocation ratio, at least 79 participants were required in each group, for a total of 158 participants. After allowing for approximately 10% invalid or missing data, the minimum required sample size was approximately 176 participants.

Normality of continuous variables was assessed using the Shapiro–Wilk test. Normally distributed data are presented as mean ± standard deviation and were compared using the independent-samples t test. Non-normally distributed data are presented as median (Q1, Q3) and were compared using the Mann–Whitney U test. Categorical variables are presented as counts and percentages and were compared using the χ^2^ test or Fisher’s exact test. Continuous variables across multiple groups were compared using the Kruskal–Wallis H test. Correlations between continuous variables and between binary and continuous variables were assessed using Spearman’s rank correlation analysis, while correlations between binary variables were assessed using the φ coefficient. Multivariable regression analyses were additionally performed using the CCDI total score as a continuous variable, adjusting for prespecified demographic (sex, age, and body weight), clinical (disease duration, aura symptoms, most common associated symptom, most common trigger, and family history of migraine), socioeconomic and health-literacy (highest caregiver education level, monthly household income, and eHEALS total score), and lifestyle- and care-related baseline covariates (sleep duration, moderate-to-vigorous physical activity, daily screen time, previous nutrition counseling, previous psychological intervention, and medication adherence); adjusted β estimates and 95% confidence intervals were reported per 10-point increase in the CCDI score.

Internal consistency of the PedMIDAS, Zarit Burden Interview, RCADS-25, and eHEALS in the present study cohort was evaluated using Cronbach’s *α*, with the corresponding coefficients provided in Supplementary Table S5.

Interrater reliability was evaluated using the intraclass correlation coefficient because all video assessment outcomes were analyzed as scores or percentage scores. The intraclass correlation coefficients for the GQS, mDISCERN, VIQI total and domain scores, and PEMAT understandability and actionability scores were calculated based on the initial independent ratings of the two primary reviewers before consensus was reached (Supplementary Table S6). All statistical analyses were performed using R software version 4.3.2. A two-sided *p* value <0.05 was considered statistically significant.

## 3 Results

### 3.1 Participant characteristics and clinical outcomes

A total of 197 children with pediatric migraine were screened, of whom 180 were included in the final analysis after 17 exclusions. According to the median CCDI score, 90 participants were assigned to the low-CCDI group and 90 to the high-CCDI group (Figure 1). The overall cohort included 92 boys (51.1%) and 88 girls (48.9%), with a mean age of 10.34 ± 1.65 years. Baseline demographic, clinical, socioeconomic, and lifestyle characteristics did not differ significantly between the two groups (all *p* > 0.05; Table 1).

**Figure 1:**
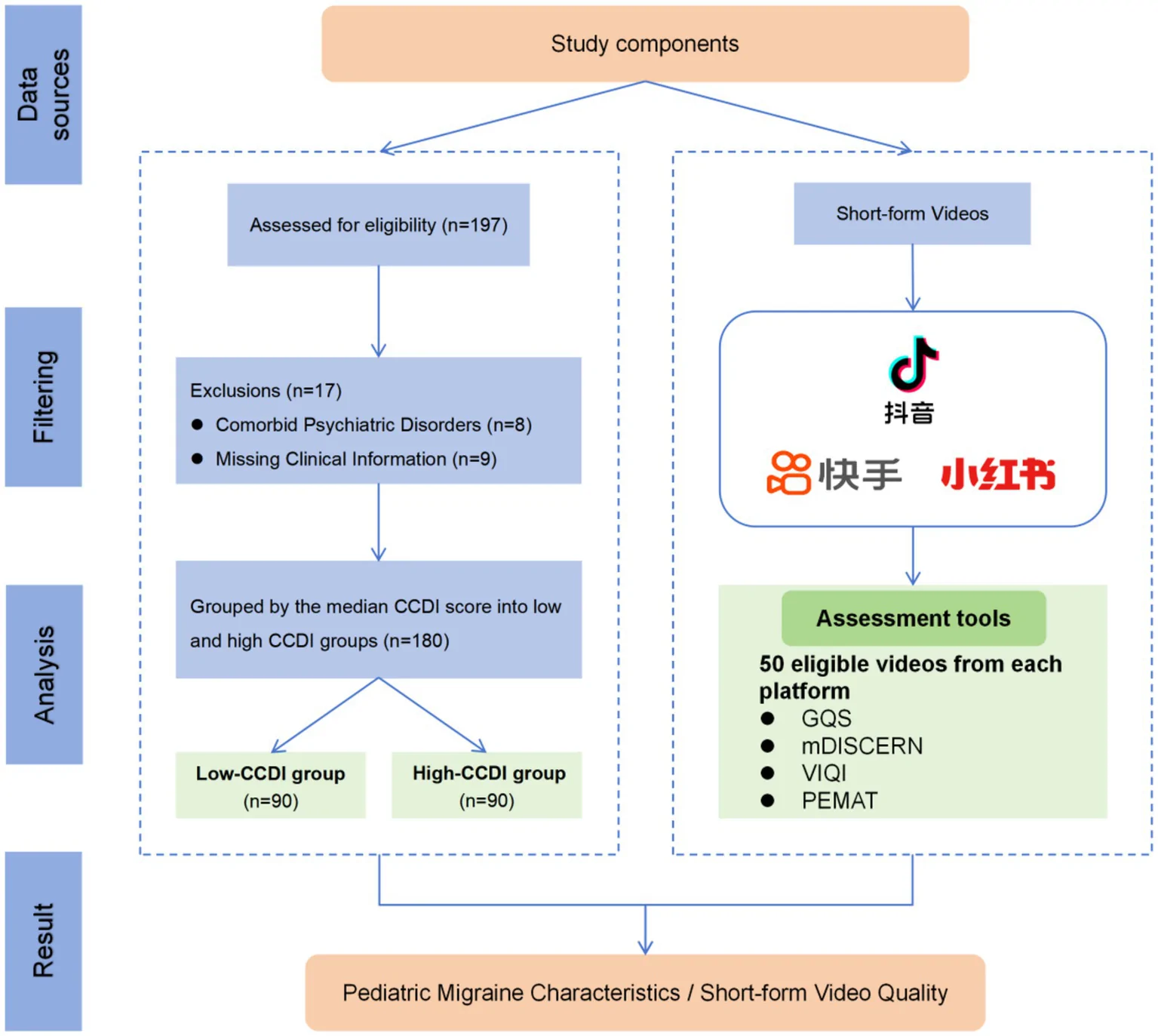
Flowchart of study design and sample selection. CCDI, Chinese Children Dietary Index; GQS, Global Quality Score; mDISCERN, modified DISCERN; VIQI, Video Information and Quality Index; PEMAT, Patient Education Materials Assessment Tool.

**Table 1:** Baseline characteristics of participants by CCDI group.

Variable	Total (*n* = 180)	Low-CCDI group (*n* = 90)	High-CCDI group (*n* = 90)	*p* value
Sex				0.551
Male	92 (51.1%)	48 (53.3%)	44 (48.9%)	
Female	88 (48.9%)	42 (46.7%)	46 (51.1%)	
Age, years	10.34 ± 1.65	10.49 ± 1.67	10.18 ± 1.63	0.207
Weight, kg	32.15 (29.10, 37.30)	32.20 (29.52, 38.50)	31.95 (28.73, 36.70)	0.302
Disease duration, months	10.00 (6.00, 13.00)	10.00 (6.00, 13.00)	9.00 (6.25, 13.75)	0.971
Aura symptoms	62 (34.4%)	30 (33.3%)	32 (35.6%)	0.754
Most common associated symptom				0.693
Nausea	89 (49.4%)	45 (50.0%)	44 (48.9%)	
Vomiting	4 (2.2%)	1 (1.1%)	3 (3.3%)	
Dizziness	39 (21.7%)	19 (21.1%)	20 (22.2%)	
Photophobia	19 (10.6%)	12 (13.3%)	7 (7.8%)	
Phonophobia	13 (7.2%)	5 (5.6%)	8 (8.9%)	
No obvious associated symptoms	16 (8.9%)	8 (8.9%)	8 (8.9%)	
Most common trigger				0.460
Excessive screen exposure	18 (10.0%)	9 (10.0%)	9 (10.0%)	
Sleep deprivation	98 (54.4%)	44 (48.9%)	54 (60.0%)	
No identifiable trigger	26 (14.4%)	15 (16.7%)	11 (12.2%)	
Academic stress	38 (21.1%)	22 (24.4%)	16 (17.8%)	
Family history of migraine	62 (34.4%)	30 (33.3%)	32 (35.6%)	0.754
Highest caregiver education level				0.198
Bachelor’s degree	66 (36.7%)	30 (33.3%)	36 (40.0%)	
Junior high school or below	11 (6.1%)	6 (6.7%)	5 (5.6%)	
College diploma	51 (28.3%)	31 (34.4%)	20 (22.2%)	
High school/technical secondary school	40 (22.2%)	20 (22.2%)	20 (22.2%)	
Graduate degree or above	12 (6.7%)	3 (3.3%)	9 (10.0%)	
Monthly household income, CNY				0.929
<5,000	35 (19.4%)	18 (20.0%)	17 (18.9%)	
≥20,000	28 (15.6%)	15 (16.7%)	13 (14.4%)	
10,000–19,999	60 (33.3%)	28 (31.1%)	32 (35.6%)	
5,000–9,999	57 (31.7%)	29 (32.2%)	28 (31.1%)	
eHEALS total score	28.27 ± 3.97	27.77 ± 3.91	28.77 ± 3.99	0.091
Sleep duration, h	8.54 ± 0.67	8.48 ± 0.65	8.60 ± 0.68	0.259
Moderate-to-vigorous physical activity, days/week	4.00 (3.00, 4.00)	3.00 (3.00, 4.00)	4.00 (3.00, 4.00)	0.122
Daily screen time, h	2.40 (1.70, 2.80)	2.40 (1.80, 3.08)	2.35 (1.70, 2.60)	0.099
Previous nutrition counseling	25 (13.9%)	15 (16.7%)	10 (11.1%)	0.281
Previous psychological intervention	26 (14.4%)	12 (13.3%)	14 (15.6%)	0.672
Medication adherence				0.400
Good	81 (45.0%)	36 (40.0%)	45 (50.0%)	
Moderate	72 (40.0%)	39 (43.3%)	33 (36.7%)	
Poor	27 (15.0%)	15 (16.7%)	12 (13.3%)	

Values are presented as mean ± SD, median (IQR), or *n* (%). CNY, Chinese yuan; eHEALS, eHealth Literacy Scale; CCDI, Chinese Children Dietary Index.

In the unadjusted group comparisons, the high-CCDI group had a significantly lower PedMIDAS score than the low-CCDI group (29.54 ± 17.20 vs. 41.96 ± 14.72, *p* < 0.001). The high-CCDI group also had significantly fewer headache days, lower pain intensity, fewer days of analgesic use, and lower ZBI total, RCADS-25 anxiety, depression, and total scores than the low-CCDI group (all *p* < 0.05; Table 2).

**Table 2:** Clinical outcomes at baseline by CCDI group.

Variable	Total (*n* = 180)	Low-CCDI group (*n* = 90)	High-CCDI group (*n* = 90)	*p* value
PedMIDAS score	35.75 ± 17.13	41.96 ± 14.72	29.54 ± 17.20	<0.001
Headache days (28 days)	8.00 (6.00, 10.00)	9.00 (8.00, 11.00)	6.00 (4.00, 8.00)	<0.001
Headache duration, h	2.80 (1.70, 3.90)	2.75 (1.62, 3.80)	2.90 (1.90, 4.00)	0.237
Pain intensity (VAS)	5.00 (4.00, 7.00)	6.00 (5.00, 7.00)	4.50 (3.00, 6.00)	<0.001
Analgesic use days (28 days)	4.00 (2.00, 6.00)	6.00 (4.00, 7.00)	2.00 (1.00, 4.75)	<0.001
ZBI total score	25.04 ± 10.51	28.21 ± 9.67	21.87 ± 10.41	<0.001
RCADS-25 anxiety score	18.98 ± 7.53	20.60 ± 7.04	17.37 ± 7.70	0.004
RCADS-25 depression score	13.69 ± 5.83	14.64 ± 5.72	12.73 ± 5.82	0.028
RCADS-25 total score	32.67 ± 10.27	35.24 ± 9.86	30.10 ± 10.08	<0.001

Values are presented as mean ± SD or median (IQR). PedMIDAS, Pediatric Migraine Disability Assessment; VAS, Visual Analog Scale; ZBI, Zarit Burden Interview; RCADS-25, Revised Child Anxiety and Depression Scale-short version; CCDI, Chinese Children Dietary Index.

The high-CCDI group had higher mean scores across all CCDI domains. The most notable between-group differences were observed for water intake, sugar-sweetened beverages, vitamin A, dietary fiber, dietary diversity, breakfast or family meals, and energy intake and sedentary behavior (Figure 2).

**Figure 2:**
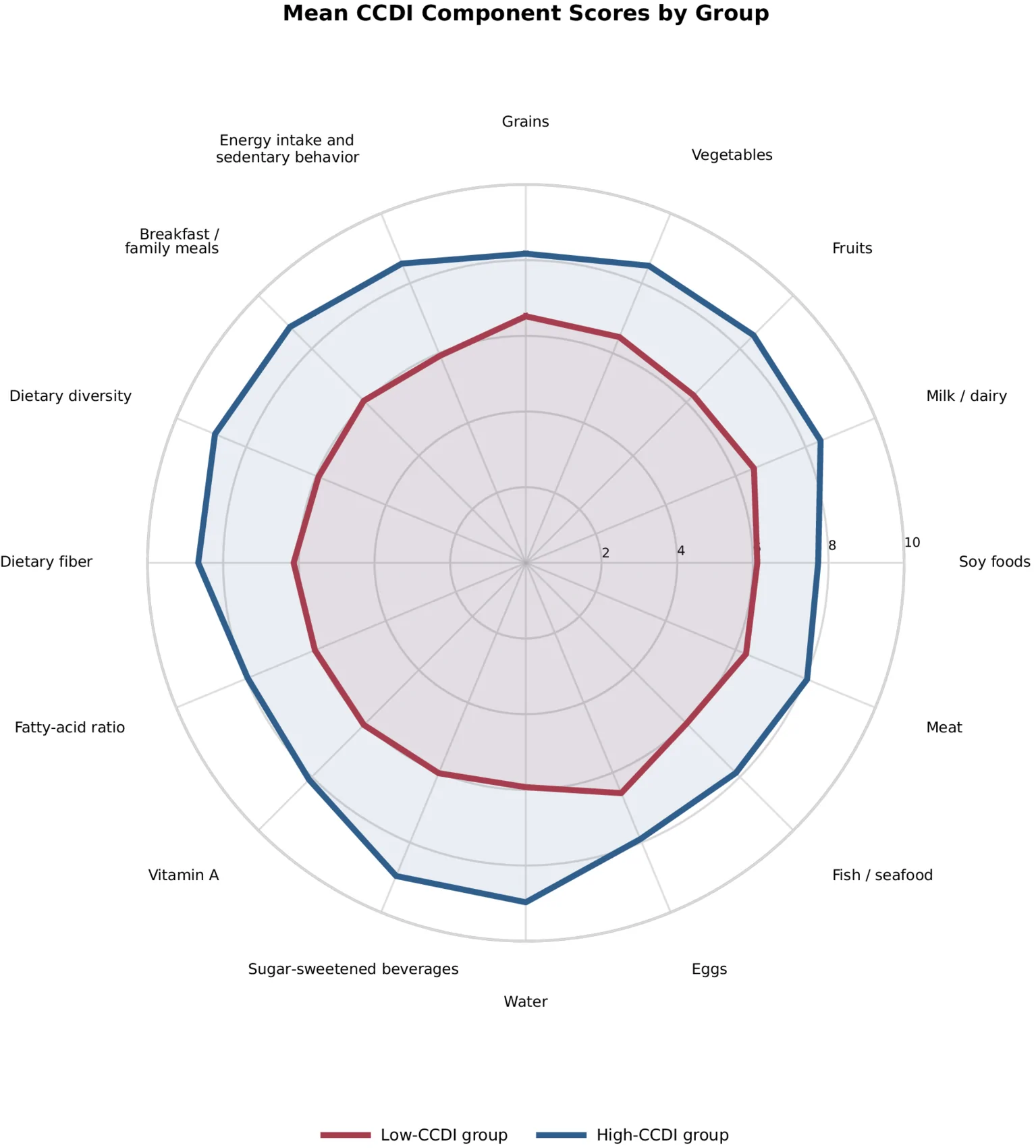
Mean CCDI component scores by group. CCDI, Chinese Children Dietary Index.

### 3.2 Caregiver use of short-form videos

The median weekly frequency of short-form video use among caregivers was 4.00 (2.00, 6.00) times, and the mean viewing duration per session was 13.84 ± 4.72 min. The most frequently searched topic was diet and lifestyle management (30.6%), followed by disease knowledge and migraine triggers (23.9%), acute headache relief methods (17.2%), medication-related information (12.8%), need for medical visit or follow-up consultation (10.0%), and psychological support and caregiver experience (5.6%) (Table 3).

**Table 3:** Characteristics of short-form video use among caregivers.

Variable	Caregivers (*n* = 180)
Weekly frequency of short-form video use, times/week	4.00 (2.00, 6.00)
Average duration per short-form video platform session, min	13.84 ± 4.72
Most common short-form video search topic	
Diet and lifestyle management	55 (30.6%)
Disease knowledge and migraine triggers	43 (23.9%)
Acute headache relief methods	31 (17.2%)
Medication-related information	23 (12.8%)
Need for medical visit or follow-up consultation	18 (10.0%)
Psychological support and caregiver experience	10 (5.6%)
Primary short-form video platform used, *n* (%)	
Douyin	96 (53.3%)
Xiaohongshu	56 (31.1%)
Kuaishou	28 (15.6%)
Subjective reliability of video content	
Reliable	99 (55.0%)
Uncertain	52 (28.9%)
Unreliable	29 (16.1%)
Verification of video advice with a physician	
Yes	62 (34.4%)
Partially verified	48 (26.7%)
No	70 (38.9%)
Adoption of video-based advice	
Yes	39 (21.7%)
Partially adopted	79 (43.9%)
No	62 (34.4%)
Satisfaction with short-form video use	
Satisfied	109 (60.6%)
Neutral	48 (26.7%)
Dissatisfied	23 (12.8%)
Behavioral responses to short-form video advice^*^	
Restricted foods because of video advice	58 (49.2%)
Used supplements because of video advice	22 (18.6%)
Used a headache–diet diary	38 (32.2%)

Values are presented as mean ± SD, median (IQR), or *n* (%). Short-form video information was available for all 180 caregivers. Subjective reliability and satisfaction were based on caregiver self-report. ^*^Behavioral responses among caregivers who adopted or partially adopted video-based advice (*n* = 118).

The most commonly used short-form video platform was Douyin (53.3%), followed by Xiaohongshu (31.1%) and Kuaishou (15.6%). Regarding subjective reliability, 99 caregivers (55.0%) considered the video content reliable, 52 (28.9%) were uncertain, and 29 (16.1%) considered it unreliable. In total, 62 caregivers (34.4%) verified video recommendations with physicians, 48 (26.7%) partially verified them, and 70 (38.9%) did not verify them. In addition, 39 caregivers (21.7%) reported adopting video recommendations, 79 (43.9%) partially adopted them, and 62 (34.4%) did not adopt them.

### 3.3 Characteristics, quality scores, and nutrition content of short-form videos

A total of 150 short-form videos were included in the analysis, with 50 videos each from Kuaishou, Xiaohongshu, and Douyin. Video duration did not differ significantly among the three platforms (*p* = 0.457). Likes, saves, comments, shares, time since posting, and number of uploader followers differed significantly across platforms (all *p* < 0.001) (Table 4).

**Table 4:** Video characteristics, quality scores, and nutrition content by platform.

Variables	Total (*n* = 150)	Kuaishou (*n* = 50)	Xiaohongshu (*n* = 50)	Douyin (*n* = 50)	*p*
Duration (s), M (Q₁, Q₃)	57.00 (39.25, 84.00)	55.00 (32.50, 78.25)	63.50 (40.50, 86.75)	58.00 (41.75, 76.50)	0.457
Likes, M (Q₁, Q₃)	700.50 (321.00, 1981.25)	982.00 (514.75, 3377.75)	74.00 (28.25, 374.00)	1143.00 (519.25, 2241.50)	<0.001
Saves, M (Q₁, Q₃)	258.50 (87.00, 824.25)	393.00 (173.25, 1353.25)	49.50 (29.00, 355.00)	354.00 (159.50, 964.75)	<0.001
Comments, M (Q₁, Q₃)	63.50 (15.25, 173.00)	74.50 (43.75, 393.00)	8.00 (1.00, 46.50)	97.50 (38.50, 224.25)	<0.001
Shares, M (Q₁, Q₃)	317.50 (77.00, 1004.50)	615.50 (224.25, 1840.00)	31.00 (9.50, 173.75)	438.00 (267.25, 1169.50)	<0.001
Time since posting (days), M (Q₁, Q₃)	554.00 (223.25, 994.50)	557.00 (178.50, 855.25)	354.00 (187.25, 595.75)	909.50 (374.25, 1330.00)	<0.001
Followers, M (Q₁, Q₃)	15500.00 (1153.75, 109000.00)	15000.00 (1296.25, 68250.00)	1203.00 (479.75, 9685.25)	274500.00 (29250.00, 768250.00)	<0.001
Uploader type, *n* (%)					0.001
Individual	23 (15.33%)	11 (22.00%)	9 (18.00%)	3 (6.00%)	
Popular science	11 (7.33%)	8 (16.00%)	3 (6.00%)	0 (0.00%)	
Physician	110 (73.33%)	31 (62.00%)	37 (74.00%)	42 (84.00%)	
Medical institution	6 (4.00%)	0 (0.00%)	1 (2.00%)	5 (10.00%)	
GQS score, M (Q₁, Q₃)	3.00 (2.00, 3.75)	3.00 (2.00, 4.00)	3.00 (2.25, 4.00)	2.00 (2.00, 3.00)	<0.001
mDISCERN score, M (Q₁, Q₃)	2.00 (2.00, 3.00)	3.00 (2.00, 4.00)	2.50 (2.00, 3.00)	2.00 (2.00, 2.00)	0.021
PEMAT-U, M (Q₁, Q₃)	68.75 (58.30, 81.81)	73.33 (63.64, 81.82)	66.70 (53.30, 83.30)	66.70 (58.73, 80.80)	0.541
PEMAT-A, M (Q₁, Q₃)	50.00 (40.00, 60.00)	60.00 (40.00, 80.00)	60.00 (40.00, 60.00)	40.00 (22.50, 50.00)	<0.001
VIQI-F, M (Q₁, Q₃)	3.00 (2.25, 4.00)	3.00 (3.00, 4.00)	2.00 (2.00, 3.00)	3.00 (3.00, 4.00)	<0.001
VIQI-A, M (Q₁, Q₃)	3.00 (2.00, 4.00)	4.00 (3.00, 4.00)	3.00 (2.25, 4.00)	2.00 (2.00, 2.00)	<0.001
VIQI-V, M (Q₁, Q₃)	2.00 (2.00, 3.00)	3.00 (2.00, 3.00)	2.00 (2.00, 3.00)	2.00 (2.00, 2.00)	0.020
VIQI-P, M (Q₁, Q₃)	3.00 (2.00, 4.00)	4.00 (2.25, 4.00)	3.00 (2.00, 4.00)	3.00 (2.00, 3.00)	0.005
VIQI-T, M (Q₁, Q₃)	11.00 (9.00, 13.00)	13.00 (10.00, 15.00)	11.00 (9.00, 13.00)	10.00 (9.00, 12.00)	<0.001
Nutrition knowledge, *n* (%)	96 (64.00%)	37 (74.00%)	37 (74.00%)	22 (44.00%)	0.001
Nutrition assessment, *n* (%)	83 (55.33%)	33 (66.00%)	31 (62.00%)	19 (38.00%)	0.010
Nutrition intervention, *n* (%)	81 (54.00%)	34 (68.00%)	26 (52.00%)	21 (42.00%)	0.031
Nutrition follow-up, *n* (%)	35 (23.33%)	11 (22.00%)	16 (32.00%)	8 (16.00%)	0.161
Special nutrition situation, *n* (%)	32 (21.33%)	11 (22.00%)	13 (26.00%)	8 (16.00%)	0.470
Guideline compliance, *n* (%)	135 (90.00%)	47 (94.00%)	43 (86.00%)	45 (90.00%)	0.411

Data are shown as median (Q₁, Q₃) or *n* (%). GQS, Global Quality Score; mDISCERN, modified DISCERN; PEMAT-U, PEMAT Understandability; PEMAT-A, PEMAT Actionability; VIQI-F/A/V/P/T, Video Information and Quality Index (Flow/Accuracy/Visual quality/Precision/Total).

Physicians were the most common uploader type, accounting for 110 videos (73.33%), followed by individual accounts (23 videos, 15.33%), popular science accounts (11 videos, 7.33%), and medical institution accounts (6 videos, 4.00%). The distribution of uploader types differed significantly across platforms (*p* = 0.001).

The median GQS was 3.00 (2.00, 3.75), the median mDISCERN score was 2.00 (2.00, 3.00), the median PEMAT understandability score was 68.75 (58.30, 81.81), the median PEMAT actionability score was 50.00 (40.00, 60.00), and the median VIQI total score was 11.00 (9.00, 13.00). Significant differences were observed across platforms in GQS, mDISCERN, PEMAT actionability, VIQI flow, accuracy, visual quality, precision, and total scores (all *p* < 0.05). PEMAT understandability did not differ significantly among platforms (*p* = 0.541).

Regarding content coverage, 96 videos (64.00%) addressed nutrition knowledge, 83 (55.33%) addressed nutrition assessment, 81 (54.00%) addressed nutrition intervention, and 35 (23.33%) addressed nutrition follow-up. Nutrition knowledge, nutrition assessment, and nutrition intervention coverage differed significantly among platforms (all *p* < 0.05), whereas nutrition follow-up coverage did not (*p* = 0.161). Special nutrition situations were addressed in 32 videos (21.33%), with no significant difference among platforms (*p* = 0.470). Overall, 135 videos (90.00%) provided nutrition recommendations that complied with clinical guidelines or evidence-based recommendations, with no significant difference among platforms (*p* = 0.411).

### 3.4 Correlation analysis

Correlation analysis of headache burden, psychological burden, and diet quality showed that the PedMIDAS score was positively correlated with headache days over the previous 28 days, pain intensity, days of analgesic use, ZBI total score, and RCADS-25 total score. The CCDI total score was negatively correlated with the PedMIDAS score, headache days over the previous 28 days, pain intensity, days of analgesic use, ZBI total score, and RCADS-25 total score. The CCDI total score was positively correlated with all CCDI domains, and most CCDI domains were positively correlated with each other (Figure 3).

**Figure 3:**
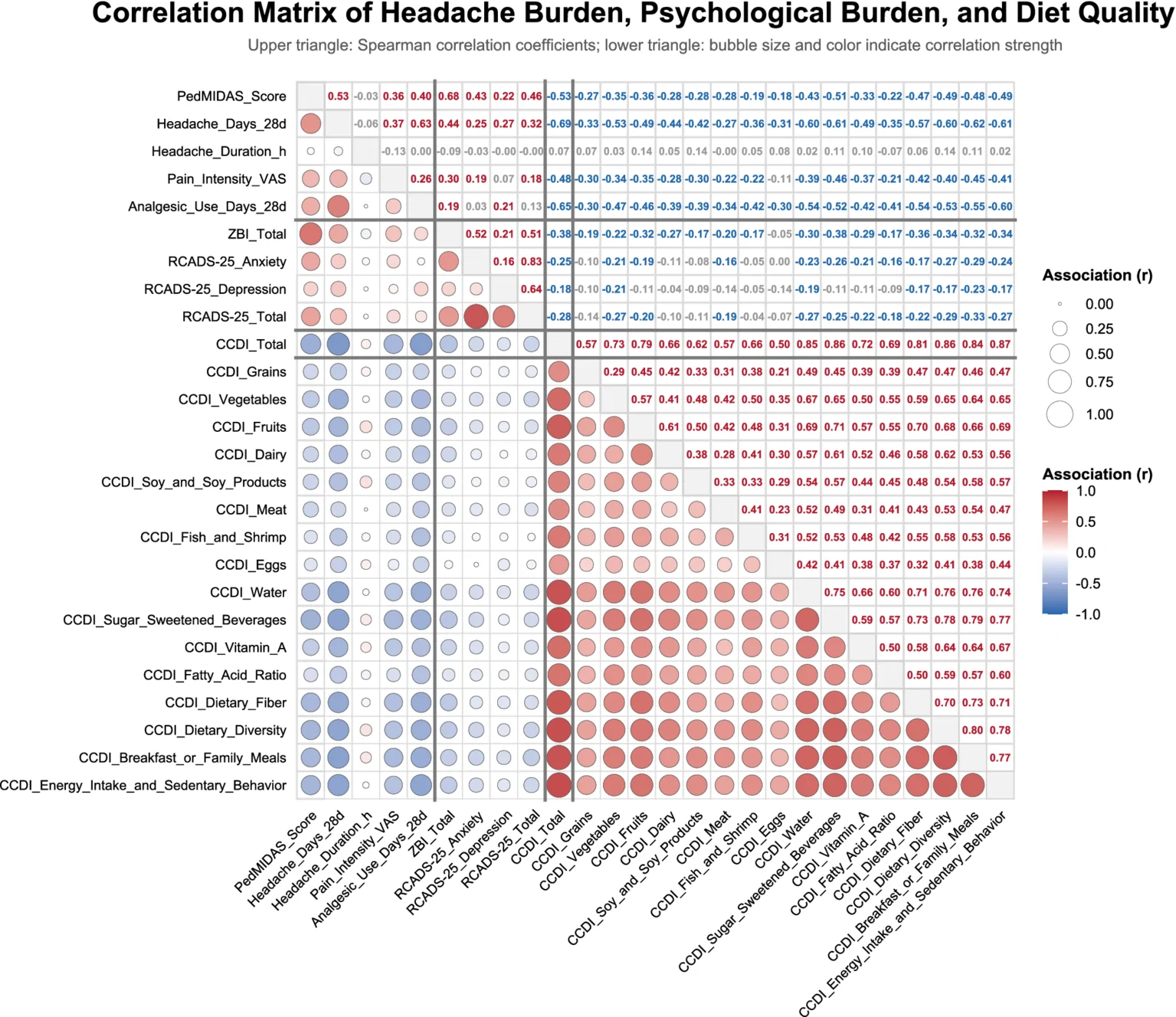
Correlation matrix of headache burden, psychological burden, and diet quality. Colored cells in the upper triangle indicate statistically significant correlations (*p* < 0.05). CCDI, Chinese Children Dietary Index; PedMIDAS, Pediatric Migraine Disability Assessment; ZBI, Zarit Burden Interview; RCADS-25, Revised Child Anxiety and Depression Scale-short version; VAS, Visual Analog Scale.

Correlation analysis of short-form video indicators showed positive correlations among likes, saves, comments, and shares. The GQS, mDISCERN, PEMAT understandability, PEMAT actionability, VIQI domain scores, and VIQI total score were generally positively correlated with each other. Nutrition knowledge, nutrition assessment, nutrition intervention, and nutrition follow-up coverage were positively correlated with some video quality scores. Overall, video dissemination metrics showed weak correlations with quality scores and nutrition content coverage (Figure 4).

**Figure 4:**
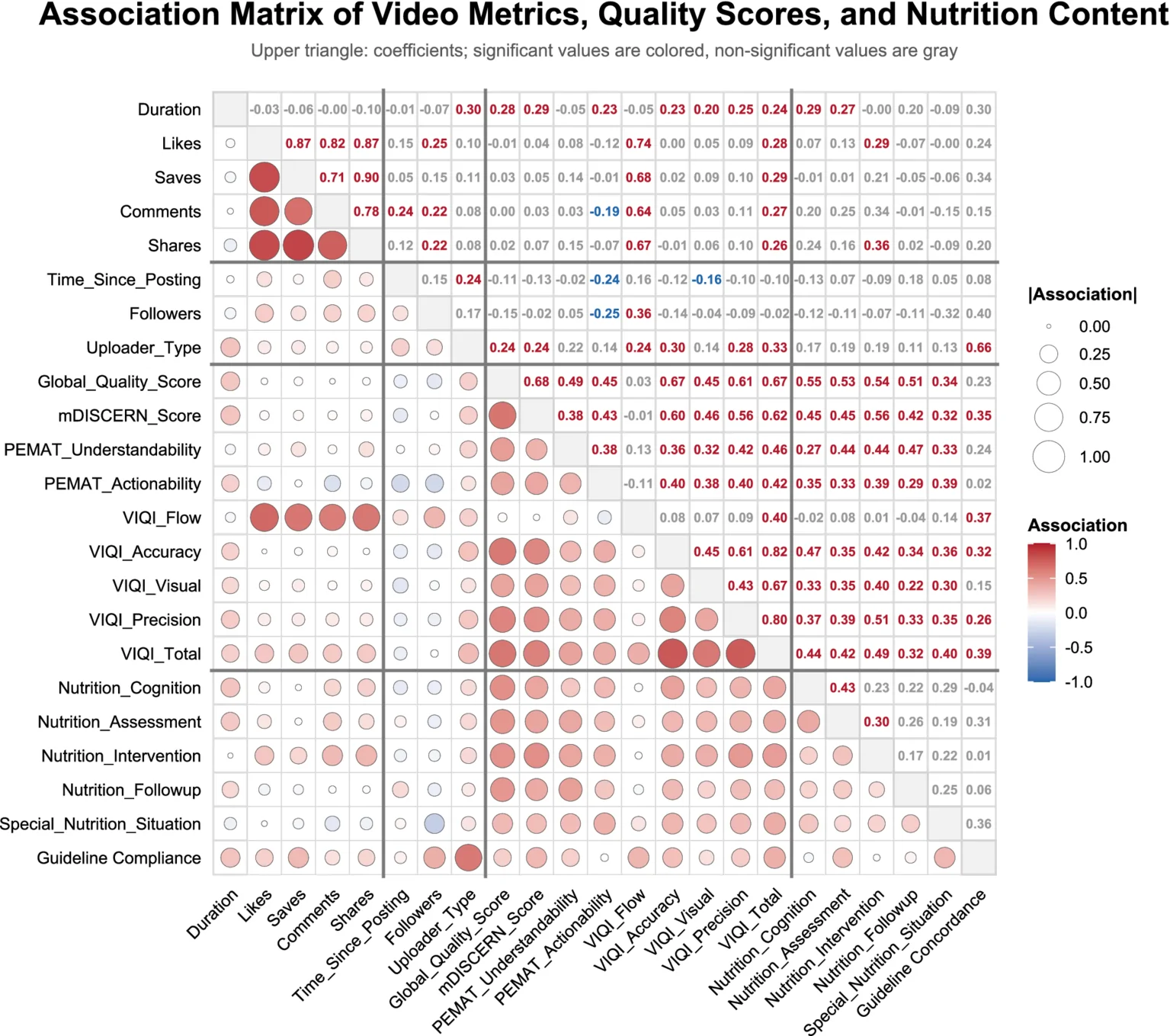
Association matrix of video metrics, quality scores, and nutrition content. Colored cells in the upper triangle indicate statistically significant associations (*p* < 0.05). GQS, Global Quality Score; mDISCERN, modified DISCERN; PEMAT, Patient Education Materials Assessment Tool; VIQI, Video Information and Quality Index.

### 3.5 Multivariable regression analysis

After adjustment for baseline covariates, each 10-point increase in the CCDI total score was associated with a lower PedMIDAS score (adjusted *β* = −2.30, 95% CI: −3.77 to −0.84, *p* = 0.002), fewer headache days over the previous 28 days (adjusted *β* = −0.96, 95% CI: −1.20 to −0.72, *p* < 0.001), lower pain intensity (adjusted *β* = −0.43, 95% CI: −0.58 to −0.29, *p* < 0.001), and fewer days of analgesic use (adjusted *β* = −0.69, 95% CI: −0.87 to −0.52, *p* < 0.001). A higher CCDI score was also associated with a lower ZBI total score (adjusted *β* = −0.94, 95% CI: −1.80 to −0.09, *p* = 0.031) (Figure 5).

**Figure 5:**
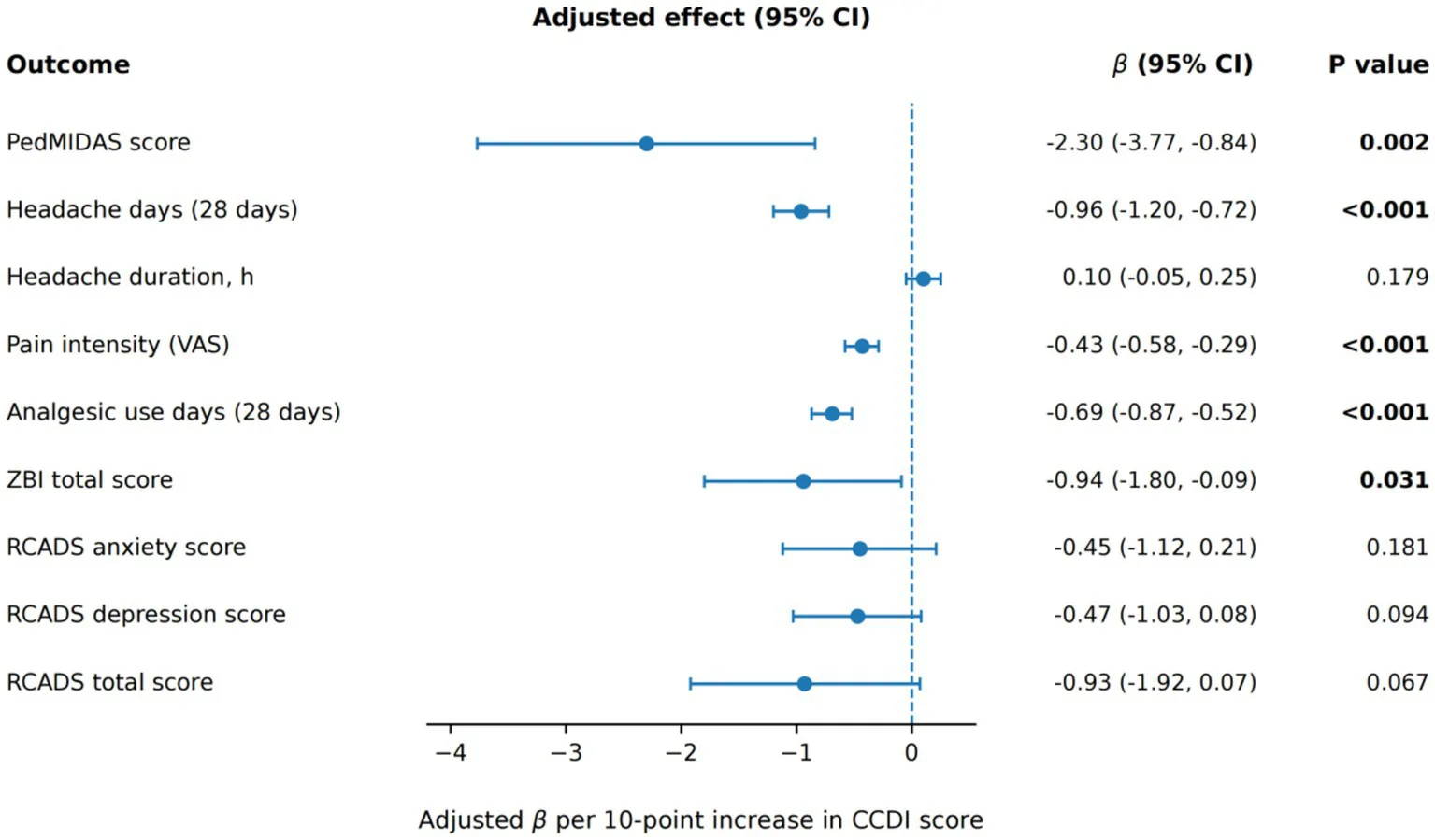
Multivariable-adjusted associations between CCDI score and clinical outcomes. Adjusted *β* estimates and 95% confidence intervals are presented per 10-point increase in the CCDI total score. Models were adjusted for sex, age, body weight, disease duration, aura symptoms, most common associated symptom, most common trigger, family history of migraine, caregiver education level, monthly household income, eHEALS total score, sleep duration, moderate-to-vigorous physical activity, daily screen time, previous nutrition counseling, previous psychological intervention, and medication adherence. CCDI, Chinese Children Dietary Index; PedMIDAS, Pediatric Migraine Disability Assessment; VAS, Visual Analog Scale; ZBI, Zarit Burden Interview; RCADS-25, Revised Child Anxiety and Depression Scale-short version; eHEALS, eHealth Literacy Scale.

## 4 Discussion

This study examined two underexplored aspects of comprehensive pediatric migraine management: diet quality and the quality of short-form video health information. Children with higher diet quality had lower PedMIDAS scores, fewer headache days and analgesic use days, lower pain intensity, lower caregiver burden, and lower anxiety and depressive symptom scores. The short-form video analysis showed a clear caregiver demand for dietary and lifestyle management information, but the reliability, actionability, and follow-up guidance of relevant videos remained insufficient. Together, these findings suggest that nutritional assessment and digital health education represent complementary aspects of pediatric migraine management, yet both remain insufficiently standardized and actionable.

Nutritional issues in pediatric migraine should be understood in terms of overall dietary patterns and daily routines rather than single food triggers alone (21). In this study, the high-CCDI group had a lower headache burden, and the associations with PedMIDAS score, headache days, pain intensity, and analgesic use days remained significant after multivariable adjustment. This is consistent with previous evidence emphasizing regular lifestyle habits and comprehensive management (22, 23). Prior studies have shown that meal skipping, dehydration, insufficient sleep, and low physical activity are associated with recurrent headaches in children and adolescents (4, 5, 24). Higher diet quality usually reflects more regular meals, greater intake of fruits, vegetables, dairy products, high-quality protein, and dietary fiber, and lower intake of sugar-sweetened beverages, energy-dense nutrient-poor foods, and caffeine. Such dietary patterns may be associated with a lower migraine burden, potentially in relation to less frequent meal skipping and dehydration, as well as differences in energy metabolism and other lifestyle factors (21, 25). However, the direction of this association cannot be determined from the present cross-sectional data. Reverse causality is also possible, as children with a lower headache burden may be better able to maintain regular meals and healthier dietary habits. Therefore, overall diet quality should be interpreted as a marker of nutritional and lifestyle patterns rather than a direct determinant of migraine control.

Specific nutrients and supplements have also received attention, but current evidence should be interpreted cautiously. A randomized pediatric trial suggested that oral magnesium oxide had uncertain preventive effects (26). High-dose riboflavin also did not show clear superiority over placebo, and pediatric evidence remains limited (6, 27). Some children and adolescents with migraine may have low coenzyme Q10 levels, and supplementation may improve headache frequency and functional impairment (28). Vitamin D, omega-3 fatty acids, and melatonin have also been studied for migraine prevention, but pediatric evidence remains limited (29–31). In our short-form video analysis, 135 of 150 videos (90.0%) provided recommendations that complied with clinical guidelines or evidence-based recommendations. However, when specific supplements such as magnesium and riboflavin were discussed, detailed guidance on dosage, duration, indications, and monitoring was not consistently provided. Thus, although the general direction of most recommendations was broadly aligned with current guidance, their practical actionability remained limited. Therefore, supplement-related content should provide clearer guidance on appropriate use, safety, and monitoring. Long-term, unsupervised, or combined supplement use without medical assessment should be avoided.

Current pediatric migraine nutrition management remains limited in both concept and implementation. In clinical practice and online education, “avoidance lists” may overemphasize the role of specific foods while ignoring the strong individual variability of food triggers. Some foods considered migraine triggers may reflect prodromal appetite changes or sensory sensitivity rather than true causes (21, 25). Broad dietary restriction without a clear individual association may be unnecessary and may reduce dietary diversity in children (32). In contrast, more common structural issues, such as breakfast skipping, inadequate hydration, insufficient intake of fruits, vegetables, and dairy products, excessive sugar-sweetened beverage intake, and low dietary fiber intake, may be overlooked in routine consultations. Similar concerns apply to unsupervised supplement use, which may occur without assessment of nutritional status, dosage, adverse effects, or drug interactions (33, 34). These findings support attention to regular meals, adequate hydration, balanced diets, individualized trigger identification, and confirmed nutritional deficiencies, although their effects on migraine outcomes require prospective evaluation.

This study also found lower caregiver burden and anxiety and depressive symptom scores in the high-CCDI group, suggesting that diet quality may be related to broader family and psychological factors. Pediatric migraine can lead to school absence, activity limitation, and reduced quality of life, and PedMIDAS reflects this disease-related functional impairment (12). Previous studies have reported psychosocial and family risks in children with migraine (35). As headache burden increases, psychological and family factors may interact. Higher diet quality may reflect a more stable family routine and stronger health management capacity and may be associated with psychological status and caregiver stress within this broader clinical context. Because this study was cross-sectional, these associations cannot be interpreted as causal. These findings support considering nutritional assessment alongside sleep, emotional status, physical activity, medication use, and caregiver burden in comprehensive evaluation.

Short-form video platforms have become an important source of pediatric migraine nutrition information for caregivers (36). Compared with traditional written materials, their accessibility, visual format, and shareability may facilitate practical demonstrations of breakfast planning, hydration reminders, headache diary use, supplement precautions, and follow-up preparation (37). Because pediatric migraine requires long-term family participation, short-form videos may extend outpatient education into daily life, and previous studies have shown that online videos are increasingly used as a supplement to patient education (38). In this study, diet and lifestyle management was the most frequently searched topic, and many caregivers fully or partially adopted video recommendations, while only 34.4% fully verified them with physicians. Given the observed association between overall diet quality and migraine burden in our clinical analysis, the quality of online nutritional information may be relevant to family dietary practices, supplement use, and management decisions. However, this study did not examine whether exposure to short-form videos directly influenced diet quality or clinical outcomes.

However, wide dissemination of short-form videos has not translated into high-quality, standardized health education. Given their role in family health decision-making (39), the quality and actionability of such information warrant particular attention. The included videos had moderate overall GQS scores, while mDISCERN and PEMAT actionability scores were relatively low. This suggests that many videos may be easy to understand but provide insufficient information on evidence sources, target users, risks and benefits, alternatives, and boundaries for seeking medical care. Their content often remains at the level of general advice such as “drink more water” and “avoid staying up late,” with limited practical guidance on hydration targets, breakfast examples, headache–diet diaries, indications for nutrition assessment, and supplement monitoring. Nutrition follow-up had the lowest coverage, suggesting a gap between online nutrition advice and the continuous assessment and adjustment required in clinical management. Social media ranking and interaction mechanisms may amplify easily disseminated content, while complex medical information is difficult to present fully in short videos (40). The weak correlations between dissemination metrics and quality scores also indicate that likes, saves, and shares mainly reflect attractiveness or platform exposure rather than medical reliability. Even videos uploaded by physicians may fail to cover dietary patterns, supplement risks, and follow-up pathways systematically because of time limits, nonstandard scripts, or insufficient nutrition professional involvement. Previous studies have similarly reported variable quality, unclear sources, and limited evidence citation in online health videos (10, 41). Quality control of short-form medical content remains a major challenge in digital health communication.

Based on these findings, pediatric migraine nutrition videos should shift from “attracting views” to “supporting decisions.” High-quality videos should reduce unsupported dietary restrictions and provide actionable guidance on regular meals, hydration, headache–diet diaries, follow-up preparation, and warning signs requiring medical attention (42, 43). Supplement education should explain the strength of evidence, indications, adverse effects, and need for medical assessment rather than presenting supplements as simple solutions for all children. Videos should also state author qualifications, evidence sources, publication dates, target age groups, and conflicts of interest, and should remind caregivers to consult physicians or dietitians before adopting dietary restrictions, supplements, or medication-related advice. Medical institutions, professional societies, and platforms could jointly develop standardized scripts and review mechanisms, with input from pediatric neurology or pain specialists, dietitians, and psychological or behavioral health professionals, to improve the accuracy, completeness, and actionability of short-form video education.

This study has several limitations. The clinical component was a single-center retrospective cross-sectional study, limiting external validity and temporal inference and introducing potential selection bias; reverse causality, whereby more severe headaches may affect dietary behaviors, cannot be excluded. The use of a dietary index developed for Chinese children and the focus on Chinese short-form video platforms may further limit generalizability across dietary cultures, healthcare systems, and digital media environments. Dietary intake, caregiver short-form video use, and adoption of video advice were self-reported and may be subject to recall bias and social desirability bias. Although multivariable analyses adjusted for available demographic, clinical, socioeconomic, and lifestyle factors, residual confounding from unmeasured factors such as pubertal status, sleep comorbidities, prophylactic medication use, and family health behaviors cannot be excluded. Short-form video content is strongly influenced by search time, region, platform algorithms, keywords, and account activity, so the findings reflect content quality within a specific search window. This study evaluated video quality and caregiver use but did not directly measure changes in caregiver knowledge, dietary behavior, or child headache outcomes after video viewing. Although mDISCERN, GQS, VIQI, and PEMAT are widely used for health information evaluation, their suitability for highly compressed and visual short-form videos remains limited. Future studies should develop evaluation frameworks better suited to short-form nutrition education for pediatric chronic diseases.

## 5 Conclusion

In summary, this study highlights the relevance of assessing overall diet quality and appropriate supplement use beyond single-food avoidance in pediatric migraine. Higher diet quality was associated with lower headache burden, caregiver burden, and emotional symptoms, supporting the relevance of nutritional assessment within comprehensive pediatric migraine care. Short-form video platforms have become an important channel for caregivers to obtain dietary and lifestyle information on migraine, with advantages in rapid dissemination, visual presentation, and relevance to family settings. However, current videos remain limited in reliability, evidence presentation, individualized guidance, actionability, and follow-up advice. Future prospective studies are needed to evaluate whether improving diet quality can improve pediatric migraine outcomes. Closer integration of clinical nutritional assessment with standardized short-form video education involving physicians, dietitians, and digital platforms may improve the consistency and actionability of nutrition communication for pediatric migraine.

## Data Availability

The raw data supporting the conclusions of this article will be made available by the authors, without undue reservation.
