# Supplementary material for: Diet quality in pediatric migraine and the educational quality of related short-form videos: a cross-sectional study and content analysis
Source: Front Nutr. 2026 Sep 21;13:1934926. doi: 10.3389/fnut.2026.1934926 (PMC13634853; doi:10.3389/fnut.2026.1934926)
Supplement: Supplementary file 1 [file Supplementary_file_1.zip › Supplementary Table 1 to 3 and 5 to 6.docx]

**Tables of contents**

Table S1. The Modified DISCERN (mDISCERN) quality criteria.

Table S2. The Global Quality Score (GQS) quality criteria.

Table S3. The Video Information and Quality Index (VIQI) quality criteria.

Table S4. The Patient Education Materials Assessment Tool (PEMAT).

Table S5. Internal Consistency Reliability of Study Measures.

Table S6. Interrater Reliability of Video Quality and Patient Education Assessment Scores.

**Supplementary Table S1** The Modified DISCERN (mDISCERN) quality criteria.

**Reliability Score**

1. Is the video clear, concise, and understandable?

2. Are valid sources cited?

3. Is the content presented balanced and unbiased?

4. Are additional sources of content listed for patient reference?

5. Are areas of uncertainty mentioned?

(1 point for answer ‘yes’, 0 point for answer ‘no’)

**Supplementary Table S2** The Global Quality Score (GQS) quality criteria.

| **Score** | **Item features** |
| --- | --- |
| 1 | Poor quality; poor flow of the videos; most information missing; not at all useful for patients |
| 2 | Generally poor quality; some information listed, but many important topics missing; of very limited use to patients |
| 3 | Moderate quality; suboptimal flow; some important topics adequately discussed, but other information poorly discussed; somewhat useful for patients |
| 4 | Good quality and generally good flow; most of the relevant information listed, but some topics not covered; useful for patients |
| 5 | Excellent quality and flow; very useful for patients |

**Supplementary Table S3** Video Information and Quality Index (VIQI)

| **Score** | **Item features** |
| --- | --- |
| 1-5 | VIQI 1: Information Flow |
|  | VIQI 2: Information Accuracy |
|  | VIQI 3: Visual/Production Quality |
|  | VIQI 4: Precision (Coherence between the video title and content) |

The score of each question ranges from 1 to 5. The total score is 4 to 20

**Supplementary Table S5** Internal Consistency Reliability of Study Measures

| **Scale / Subscale** | **Cronbach's α** |
| --- | --- |
| eHEALS | 0.887 |
| PedMIDAS | 0.821 |
| ZBI | 0.903 |
| RCADS Anxiety Subscale | 0.914 |
| RCADS Depression Subscale | 0.869 |
| RCADS Total Scale | 0.928 |

Abbreviations: eHEALS, eHealth Literacy Scale; PedMIDAS, Pediatric Migraine Disability Assessment; ZBI, Zarit Burden Interview; RCADS, Revised Child Anxiety and Depression Scale.

**Supplementary Table S6** Interrater Reliability of Video Quality and Patient Education Assessment Scores

| Rater pair | Stage | Assessment score | IRR statistic | Value | 95% CI | Final score used in analysis |
| --- | --- | --- | --- | --- | --- | --- |
| Researchers | Preconsensus | GQS score | ICC | 0.81 | 0.71–0.89 | Consensus score |
| Researchers | Preconsensus | mDISCERN score | ICC | 0.82 | 0.72–0.90 | Consensus score |
| Researchers | Preconsensus | VIQI total score | ICC | 0.86 | 0.79–0.91 | Consensus score |
| Researchers | Preconsensus | VIQI flow score | ICC | 0.75 | 0.62–0.85 | Consensus score |
| Researchers | Preconsensus | VIQI accuracy score | ICC | 0.82 | 0.71–0.90 | Consensus score |
| Researchers | Preconsensus | VIQI visual quality score | ICC | 0.79 | 0.70–0.88 | Consensus score |
| Researchers | Preconsensus | VIQI precision score | ICC | 0.80 | 0.60–0.89 | Consensus score |
| Researchers | Preconsensus | PEMAT understandability (%) | ICC | 0.89 | 0.82–0.94 | Consensus score |
| Researchers | Preconsensus | PEMAT actionability (%) | ICC | 0.85 | 0.74–0.91 | Consensus score |

Abbreviations: CI, confidence interval; GQS, Global Quality Score; ICC, intraclass correlation coefficient; IRR, inter-rater reliability; mDISCERN, modified DISCERN; PEMAT, Patient Education Materials Assessment Tool; VIQI, Video Information and Quality Index.
